# The use of antenatal care in two rural districts of Upper West Region, Ghana

**DOI:** 10.1371/journal.pone.0185537

**Published:** 2017-09-28

**Authors:** Joshua Sumankuuro, Judith Crockett, Shaoyu Wang

**Affiliations:** School of Community Health, Faculty of Science, Charles Sturt University, Orange, New South Wales, Australia; University of Washington, UNITED STATES

## Abstract

**Background:**

Despite decades of implementation of maternity healthcare programmes, including a focus on increasing the use of antenatal care (ANC) and concomitant birth preparedness and complication readiness (BPCR), the uptake of ANC continues to be below expectations in many developing countries. This has attendant implications for maternal and infant morbidity and mortality rates. Known barriers to ANC use include cost, distance to health care services and forces of various socio-cultural beliefs and practices. As part of a larger study on BPCR in rural Ghana, this paper reflects on the use of ANC in the study areas from rights-based and maternal engagement theoretical perspectives, with a focus on the barriers to ANC use.

**Methods:**

Mixed methods approach was adopted to collect data from 8 study communities from individual in-depth interviews with 80 expectant mothers and 13 health care professionals, and 24 focus groups comprising 240 community members. The qualitative data followed a thematic analytical method, while the quantitative data was analysed using descriptive statistics.

**Results:**

The average number of ANC visits were 3.34±1.292, and the majority of expectant mothers (71.3%) enrolled for ANC at the 8^th^ week or later, with the longest delay recorded at the 6^th^ month of gestation. Traditional norms significantly influenced this delay. Likewise, overall use of ANC during pregnancy was shaped by cultural factors related to perceptions of pregnancy, gender-based roles and responsibilities and concerns that ANC would result in an overweighed baby and culturally inappropriate delivery at a health care facility.

**Conclusion:**

Greater understanding of the sociocultural barriers to ANC is essential if proposed changes in community-specific health education programs are to facilitate early commencement and increased use of ANC.

## Introduction

The World Health Organisation (WHO) notes how a woman dies in every two minutes from pregnancy or related causes, the world over [1]. Forty percent of the countries with high levels of maternal mortality are in sub-Saharan Africa (SSA), and women in the region face 15 times the risk of dying from pregnancy and childbirth situations compared to women in developed countries [2]. Statistics indicate that many maternal deaths occur because of preventable causes such as haemorrhagic shock, infections, obstructed labour, and hypertensive disorders in pregnancy and abortion in rural northern Ghana [3]. Antenatal care (ANC) has been a valuable tool in reducing maternal deaths through; 1) early identification and management of obstetric complications such as pre-eclampsia, 2) tetanus toxoid immunisation, 3) intermittent preventive treatment for malaria during pregnancy (IPTp), and 4) identification and management of infections including HIV, syphilis and other sexually transmitted diseases (STDs) [4–6]. Therefore, the WHO recommends up to eight ANC visits for all expectant mothers in developing countries [4–8].

Even though the strategy has proved useful in addressing many problems in pregnancies and ensuring safe births, profound barriers to ANC utilisation continue to exist in many locations due to the interactions of socioeconomic influences (such as accessibility, cost) [9, 10], health service-related factors (such as lack of trained staff and other resources) [11, 12], and a diverse array of cultural beliefs and practices [3, 6].

For example, in Tigray Zone, Ethiopia, many expectant mothers had no knowledge of the benefits they would derive from utilising skilled maternity care; this lack of health literacy, combined with mockery, shame and stigmatisation from the family and community if they sought ANC, resulted in the absence of ANC uptake[12, 13]. In some communities of the Upper West Region (UWR) the expectant mother had to gain approval from the husband (and in some locations, permission from the community) before seeking ANC at a health facility [14, 15], and a man accompanying their wife to ANC was seen as a violation of cultural norms [14]. In these locations, expectant mothers could register for ANC but fail to follow-up or implement therapeutic interventions [4], and preference for home birth took precedence over ANC [16].

In another region of rural Ghana, it was observed that women who patronised ANC services were more likely to have the support of their husbands, and were more likely to be prepared and ready for birth and emergencies [13, 17].

Even where ANC is utilised, it may not necessarily provide adequate information regarding obstetric danger signs [3], indicating that the quality of ANC education is also a factor shaping maternal health outcomes. In northern Ghana, for example, only 65% of women attending ANC reported receiving education on obstetric danger signs [3, 18].

Overall, government efforts to significantly increase uptake of ANC continue to be problematic in rural Ghana [4]. These challenges call for further inquiry into the issues pertaining in the utilisation of ANC from the perspectives of hard-to-reach communities in the Upper West Region, Ghana.

## Methods

### Research setting

Nadowli/Kaleo and Daffiama/Bussie/Issa (DBI) districts have a total population of 94,388, comprising 61,561 and 32,827 in the respective districts. Both districts have a sex-disaggregated split of 48% males and 52% females [19, 20]. The proportion of illiterate males and females 11 years and over in the Daffiama/Bussie/Issa district were 51.8% and 63.0%, respectively. In the Nadowli/Kaleo district, the illiteracy rates were 40.2% and 56.2% for males and females 11 years and over, respectively [19, 20].

The majority of inhabitants in the two districts engaged in subsistence farming, with maize, millet, guinea corn and groundnuts as the major food crops [19–22]. There is only one tarred road in the region, the Economic Community of West African States (ECOWAS) road, which links the nation’s capital through Kaleo, Nadowli to Burkina Faso. Bicycles and motorbikes were the dominant means of transport, including during labour and obstetric complications [19, 20, 22]. Feeder roads link the communities, but the majority were not motorable during the rainy season. Even the motorable roads in the dry season had many hazards to vehicles and motorists [19–22].

### Research design

The study adopted the pragmatic research paradigm underpinned by mixed qualitative and quantitative methods to explore access and utilisation of antenatal care (ANC) as a component of a broad study in rural terrain. The survey, focus groups discussions (FGDs) and in-depth interviews (IDIs) were the techniques adopted to collect relevant data for the study. These were informed by the complexity of issues about maternal and neonatal health outcomes in rural communities and low ANC follow-up [4]. Qualitative interviews and group discussions were the appropriate means to obtain community perspectives on maternal and neonatal healthcare including the challenges on ANC services uptake, while the quantitative survey offered expectant mothers the opportunity to provide information on issues identified in the ANC literature.

### Community selection

The eight study communities (four in Nadowli/Kaleo and four in Daffiama/Bussie/Issa districts) were purposively selected as communities with varying levels of access to preventative and curative healthcare services, including to the highest referral health facility (Nadowli Hospital). Health Centres served four of the communities, and CHPS compounds served the remainder; these are the lower two tiers of health care delivery by Ghana Health Service.

### Participant sampling and recruitment

To obtain data saturation, a mix of purposive and key informant sampling procedures were employed to obtain 333 participants, constituting forty participants from each of the eight communities (10 expectant mothers and 30 community residents per community), in addition to 13 health care staff Table 1.

**Table 1:** Composition of participants groups.

Participants	Age range (years)	Number	Data type	Sex disaggregation	No. of Communities
Opinion leaders	18–59	80	Qualitative data	22 females; 58 males	8
Non-pregnant women	18–59	80	Qualitative data	All females	8
Youth	18–35	80	Qualitative data	40 females; 40 males	8
Expectant mothers	18–49	80	Quantitative	All females	8
Healthcare staff	25–59	13	Mixed	11 females; 2 males	10 (8 communities, two district health administrations)

#### Expectant mothers

The Women in Fertility Age (WiFA) data (reproductive age range of 15–49) and the number of deliveries and projected deliveries for the period 2012 to 2014 were obtained from the Health Directorates. This formed the basis for deciding 10 expectant mothers (those in gestational age of second and third trimesters excluding the ninth month) would be surveyed in each community.

The list of all expectant mothers receiving ANC and meeting the selection criteria was obtained from the health facility. Pregnant women not using ANC were also identified in each of the communities. A random selection of 10 mothers of different age groups and parity was made in each community, ultimately resulting in the recruitment of 67 mothers who were receiving some level of ANC and 13 mothers not in receipt of ANC.

#### Focus groups

In collaboration with the local elected community representative, 10 opinion leaders (age range 18–59), 10 non-pregnant women with previous birth experiences (age range 18–59) and 10 youth group leaders were purposively selected based on their respective roles as ‘key informants’ in each of the eight communities. A focus group of each type was run in each community [23]. Segmenting participants by age and sex provided all those involved with the freedom to openly express their views and to gain a cross-section of opinion. The sample sizes were pre-determined to ensure data saturation in the themes [23].

#### Health professionals

After support was received from the district assemblies and directors of health services, contacts were made with two district directors of health, eight heads of health facilities from the eight research sites, and three non-antenatal care unit nurses from the eight communities, all of whom were asked to participate in in-depth interviews.

### Research instruments

The study used three different but related tools. A survey conducted in ‘Dagaare’ (the local language) comprising multiple closed-ended questions and four open-ended questions was used for the expectant mothers. This focused on understanding the mother’s opinions on birth preparedness and complication readiness, ANC attendance, previous pregnancy experiences, family involvement and support during pregnancy and childbirth, perceived causes of morbidities and mortalities and socioeconomic issues impacting on safe pregnancy and childbirth.

An interview schedule containing structured and unstructured questions were used with health professionals and encompassed staffing and logistical capacities to provide quality ANC services, health care financing issues and preparedness for birth and complications. A semi-structured discussion guide was utilised in FGDs, enabling the collection of community views on BP/CR interventions, the causes of maternal and neonatal morbidities and mortalities, sociocultural beliefs and practices impacting the use of maternal and newborn health services, and any issues emerging from expectant mothers’ interviews.

### Data collection

Quantitative data were collected first, before the semi-structured FGDs and IDIs. Interviews with pregnant women lasted between 45 minutes to one hour, while the IDIs and FGDs lasted between 1 and 1.5 hours and ended when no new issues seemed to arise, thus, when data saturation was attained.

The FGDs were conducted at convenient venues for participants in ‘Dagaare’. Accompanying the first author during the FGDs was a senior researcher in community-based approaches to health care studies and also a local, who acted as a scribe and picture-taker [24].

The IDIs with healthcare professionals were conducted in the English language.

### Data processing

All interviews and focus group discussions were tape-recorded with the informed consent of the participants. Audio tapes were replayed to the hearing of participants to ensure issues recorded corresponded with the research questions and participant views. To facilitate data accuracy, we first transcribed audio-recorded tapes of the FGDs and surveys in “Dagaare”, and then translated the transcripts into the English language. Interviews with healthcare staff were transcribed in the English Language.

### Data analysis

Analysis of interview and focus group discussions (FGDs) was an iterative process which began in the field. After each interview, notes were made regarding: a) notes about body language or other things not captured by the recording; b) emerging opinions from the participants and how they could be noted and applied to other interviews; c) what went well or not-so-well; d) what should be done differently in future interviews; e) physical observations of health facilities, surface nature of roads, interactions among participants and nurses as compared to feedbacks and responses to the interviews. During the qualitative data collection (interviews with “other nurses” and FGDs sessions), an analytical process shaped the ongoing data collection. This interim analysis accorded the researcher the advantage to fine-tune some questions to pursue emerging issues of inquiry in further depth. Crucially, it also enabled the field interviews and discussions to explore cases that identified behaviours and sociocultural norms that run counter to current and emerging opinions on antenatal care.

SPSS (version 20) and NVivo (version 7.5) were used to analyse the quantitative and qualitative data, respectively. Text analytical categories and themes related to “causes of complications, home births, causes of morbidities and mortalities, risk factors of poor health outcomes” were deductively formulated in correlation with quantitative variables. The *priori* codes were developed from literature and experience. Emergent codes were developed from the transcripts, direct notes and field reflection to complement similar variables identified in the quantitative data “S1 File”. Textual data codes from each of the 24 FGDs and 13 IDIs were used to validate relative impacts of each issue on maternal and neonatal health care and health outcomes. Computerised coding and repeated manual searches were conducted to ensure all salient texts, including participant quotes, were identified regarding *priori* major and sub-themes in the quantitative data.

To understand the profoundly complex issues facing maternal and neonatal health, the study draws on maternal engagement theory [25] and rights-based approach to decision-making [26], both of which are founded on feminist principles and strongly orientated towards the well-being of women and children [26, 27].

### Ethical considerations

The research forms part of a Doctoral project sponsored by the Government of Ghana through its Education Trust Fund. Charles Sturt University Human Research Ethics Committee approved the conduct of the study (Protocol number: 2016/013). Letters of support were obtained from the Regional and District Health Directorates “S2, S3 and S4 Files”.

The principal researcher hails from the study region, and this facilitated establishing contacts with research participants and research communities and increasing the overall participant response rate. However, the selection of the research participants was strictly based on the research procedures and not on any merit or favour.

## Results

### Basic demographic characteristics of expectant mothers

The gestation of pregnancies was randomly a 50–50 split between the second (40 participants) and third trimesters (excluding ninth month) (40 participants).

The demographic characteristics of expectant mothers are noted Table 2. The clear majority (93.8%) were married, 53.8 percent had never attended school, while 43.8 percent engaged in farming for a livelihood. Almost all of the pregnant women involved in strenuous work. Twenty percent were in their first pregnancies Table 2.

**Table 2:** Basic demographic characteristics of expectant mothers (N = 80).

Characteristic	Number	Percent (%)
**Highest educational level**		
Senior high school	10	12.5
Junior high school	16	20.0
Primary school	11	13.8
Never attended	43	53.8
**Current marital status**		
Married	75	93.8
Divorced	1	1.3
Co-habitation	4	5.0
**Gravidity**		
One	16	20
Two	12	15
Three	13	16.3
Four	16	20
≥Five	23	28.8
**Current job for livelihood**		
Housewife	2	2.5
Farmer	35	43.8
Wood logging/charcoal burning	9	11.3
Local wine brewing	17	21.3
Other mixed activities	17	21.3

### Antenatal care patronage

**Fig 1:**
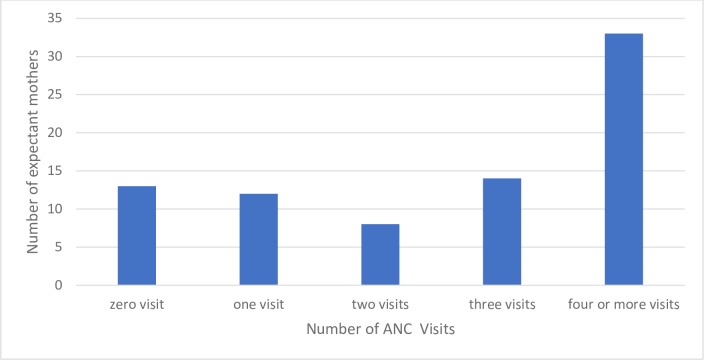
Number of ANC visits in present pregnancy.

**Fig 2:**
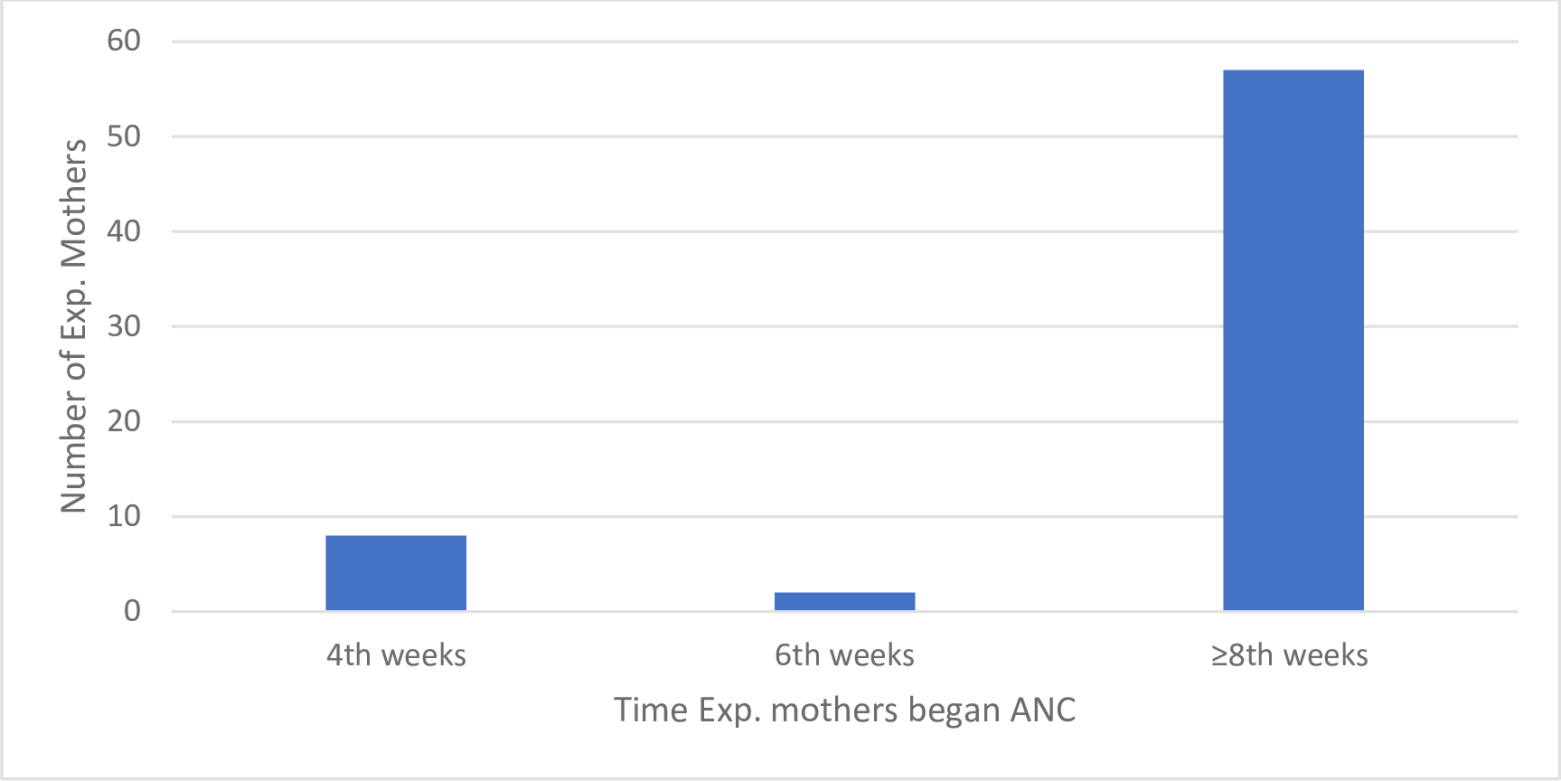
Time of first antenatal care visit.

Expectant mothers were asked about the extent of their ANC attendance during their current pregnancy. Whilst a large number of pregnant women completed four or more visits (n = 33, 41.3%), some had one visit (n = 12, 15%), two (n = 8, 10%) and three (n = 14, 17.5%). Approximately 16.3% (n = 13) had not yet commenced ANC Figs 1 and 2. That is, 58.7% of expectant mothers had attended fewer than four visits at the time of interview.

### The time expectant mothers commenced antenatal care

The majority of pregnant women (n = 57, 71.3%) enrolled for ANC at the 8^th^ week or later Fig 2, with the longest delay recorded at sixth month. Thirteen expectant mothers had not commenced ANC at the time of data collection, two of whom were prime mothers and two of whom were multipara mothers in their sixth and seventh months of gestation respectively.

### Perceptions of antenatal care

#### From the perspective of health care professionals

Perceptions of ANC from the standpoint of health care providers is encapsulated in the following quote:

When pregnant women register for ANC, they are encouraged to bring family or community members to donate blood so it can be stored at the blood bank pending any emergency. Expectant mothers are also asked: to save money towards emergency referrals, complications and normal childbirth … purchase clothes, baby clothes, and clean rags and keep with them always …. or keep the birth kit within reach of family members …. take their NHIS card with them always so no cost will be involved during delivery…the fifth aspect of the BPCR education is to walk along with their ANC card so that she can receive care at any nearby healthcare facility…… [IDIs, other nurses].

It can be observed from this quote that, from the perspective of health care providers, ANC has a strong focus on birth preparedness and complication readiness, in addition to monitoring the progress of the pregnancy and well-being of the expectant mother.

#### Perceived benefits of ANC services by expectant mothers

Pregnant women were asked to identify possible benefits they would gain from ANC. All the pregnant women identified at least one benefit of utilising ANC, with the majority perceiving ANC as a programme for recognition and management of pregnancy and related complications (n = 46, 57.5%). Being able to receive preventive measures such as immunisations throughout the period (n = 22, 27.5%) was also important Table 3.

**Table 3:** Perceived benefits to ANC utilisation by all expectant mothers.

Category	Number	Percent (%)*
Perceived benefits of ANC		
Health pregnancy classes	5	6.3
Preventive measures including immunisations	22	27.5
Health education and promotion for you and family	2	2.5
Recognition and management of pregnancy and related complications	46	57.5
Prepare us emotionally and physically for the pregnancy	5	6.3

* out of 80 expectant mothers

Overall, the majority of women understood that early detection of risks and administration of relevant medicines and laboratory tests and ultrasound scans have considerable positive benefits for safe pregnancy, childbirth and the puerperal period, particularly if commenced in the first month of gestation:

We are usually asked to commence ANC in the first month of pregnancy so that the medications can strengthen us to progress safely and have safe childbirth [FGDs, non-pregnant women, Charikpong].

### Barriers to ANC early uptake and continuity

#### Barriers identified by expectant mothers

Despite the perceived benefits of ANC, expectant mothers gave many reasons for not completing a minimum of four ANC visits, including distance to ANC venue (33.3%), transport difficulties (11.9%), cultural influence (2.4%) and social reasons (2.4%) and “not far into pregnancy (11.9%)” Table 4. No expectant mother identified the family as a barrier to the inability to commence ANC early or complete the follow-ups Table 4.

**Table 4:** Barriers to utilisation of ANC per expectant mothers.

Reasons for less than four ANC visits	Number*	Percent (%)
Distance to ANC venue	14	33.3
Cultural reasons, e.g. family objections	1	2.4
Transport challenge	5	11.9
Social reasons, e.g. funeral	1	2.4
not far enough along in pregnancy	5	11.9
Other	16	38.1
**Total**	**42**	**100.0**

Note

* these numbers are not mutually exclusive of the expectant mothers

#### Assistance to attend ANC

While the pregnant women identified that they usually attended ANC on their own, or with another expectant mother Table 5, they did not see this as a barrier to using ANC.

No, here we do not do those things! We are not used to seeking care with them (pregnant women) [a man, FGDs, opinion leaders, Woggu].

Sometimes, because of the farm work, instead of the man accompanying us for ANC, we are happy to go alone while he continues with the agricultural work [FGDs, non-pregnant women, Naro/Korinyiri].

**Table 5:** Family support during pregnancy.

Category	Number of expectant mothers	Percent (%)	
**Family involvement during pregnancy**			
Yes	39		48.8
No	41		51.3
Support to a health facility during pregnancy* (n = 80)			
Husband/partner	8		10.0
Mother-in-law	5		6.3
Close relatives	10		12.5
Others/alone	57		71.3

Note:

* means providing financially and basic needs, accompanying during care-seeking

If the expectant mother was referred to the hospital from a health centre or CHPS compound for ANC related matters, it was more likely that a family member would accompany her.

However, when she is on referral, we assist in reaching the hospital. Normal ANC, we do not support or accompany them. We are not used to going to the clinic with them [FGD, opinion leaders, Jang].

Generally, farmers do not accompany expectant mothers for routine ANC. Husbands are compelled to visit the clinic with pregnant women when she is sick at night or on referral to Nadowli hospital [FGD, opinion leaders, Woggu].

#### Distance and transport challenges

Following ANC registration at the health centre or CHPS compounds, expectant mothers are referred to Nadowli hospital for laboratory investigations. However, the distance between the centres, compounds and the hospital, coupled with the poor surface nature of the roads and scarce vehicular transport makes attendance difficult:

When an expectant mother registers for ANC, we are first referred to Nadowli hospital for laboratory tests and scans which some mothers refuse to go, because of the distance and cost of reaching Nadowli. Other times, if the husband has no motorbike and she happens not to have lorry fare to transit through Wa or Sombo to Nadowli, she would not receive laboratory investigations on time leading to late uptake of care. Pregnant women do foot for more than five kilometres to Sombo to join public transport (“trotro”) to Nadowli. Alternatively, others make two transits through Wa to Nadowli [FGDs, non-pregnant women, Naro/Korinyiri].

#### “Not far enough along in the pregnancy.”

It was common in the study locations to commence ANC late.

Some will be there three months, four months before they will come for ANC card and when you ask, they do not have any reason. They were only waiting to be sure that they were pregnant. Some will be there, and before they come for ANC they are 28 or 32 weeks, and that is third-trimester registration [IDIs, midwife, Health facility in-charge, Bussie].

#### The need for pregnancy announcement or cleansing rite

Community members in Bussie, Woggu, Jimpensi/Kenkelley, Charikpong and Jang believe that ANC attendance cannot commence until after pregnancy “announcement” or “cleansing” rite for prime mothers who were married is carried out. The essence is to prevent miscarriage and complications. Further, in Charikpong, Jimpensi/Kenkelley and Naro/Korinyiri, the practice was repeated in every pregnancy in some families.

We do it anytime we discover she has conceived. Otherwise, when the public discusses it at her hearing, she will miscarry. The practice commits it (the pregnancy) to the ancestors who prevent it from bad omens and strange spirits possession [FGDs, non-pregnant women, Bussie].

In some communities, when a woman is pregnant, they must perform certain rites before they can start receiving maternal health care or before other community members get to know. The rites are done before it is known to everyone, else, they believe she might lose the pregnancy [IDIs, DoN, female, Nadowli/Kaleo].

The rites did not include women that were not customarily married before conceiving.

#### Stigmatisation of expectant mothers

Stigmatisation (criticisms and mockery) of pregnant women by the community members was identified as a determinant for the late start of antenatal care in some cases. An expectant mother who had had 12 childbirths was found not receiving ANC despite being in her fifth month of gestation in the current pregnancy, because of stigma from the public that, “her colleagues have stopped and she is still giving birth”. She felt she was too old but needed the pregnancy because she had lost seven of the children.

Some expectant mothers deliberately refuse to seek maternal health care. Others do not want the public to know she is pregnant, particularly when there are controversies concerning her conception. They go into hiding until it is critical to their health [FGDs, non-pregnant women, Charikpong].

However, they have given birth to over 12 or more children and still get pregnant again. Hence, it is a shame to them. Hence they find it difficult coming out. Some of them too are pregnant but not by their husbands, so they try aborting and it did not work. So, they do not come for ANC [IDIs, Midwife, Health facility in-charge, Woggu].

Delays in seeking ANC is an issue for younger and or unmarried mothers, for whom the stigma is even greater:

Others hide the conception from us as family heads. They normally do not want others to know they are pregnant. Many of the expectant mothers who fail to attend antenatal care are the single ones and under-aged (less than 18) girls and school children. They keep it secretly while sometimes devising illegal means of terminating the pregnancy. Even those expectant mothers who yield to pressure to receive ANC do it at the time when they are in their second trimester [FGDs, Opinion leaders, Jang].

#### Lack of support and assistance from family (as barrier to ANC)

Even though family influence was not mentioned as a barrier to accessing ANC by expectant mothers, lack of family support was identified as a significant impediment in many focus groups and during interviews with health professionals. Some husbands/partners felt they had no responsibility to help expectant mothers to reach a suitable health facility for ANC, whether it be by providing transport, finance or company.

No, some men only impregnate and leave them to manage the pregnancy until they give birth. Some do not even take them to Nadowli to renew their health insurance and carry out laboratory tests [FGDs, non-pregnant women, Jimpensi/Kenkelley].

The habit is particularly common among farmers:

ANC can be received any day, but the moisture in the farm is available for few days, hence, let’s go to the farm. For fear of the partner, some expectant mothers comply with him and even sometimes receive few ANC visits before childbirth [FGDs, opinion leaders, Jang].

In any case, men could not see the point of attending given the refusal of some mothers to utilise ANC at a health facility:

Even, some women do not attend ANC and postnatal care, how much more we men? [FGD, opinion leaders, Woggu].

ANC was perceived as a new health initiative; hence, some farmers deny their wives’ access because they believe they could have safe pregnancy and birth outcomes without utilising maternal care from the health facility.

I have asked for money to seek maternal health care from my husband and was not given. I had to struggle to acquire money and fuelled a bike to go to Nadowli hospital for laboratory tests. My husband deliberately refused to support me nor take me to the hospital. Other men have been cited saying their mothers did not receive ANC but gave birth successfully [FGDs, non-pregnant women, Jimpensi/Kenkelley].

Sexually transmitted infections can cause pregnancy complications, but the lack of involvement of husbands/partners in ANC, with commensurate inability to treat the latter for STIs, can pose a challenge to pregnant women and nurses of treating STIs cases:

*We have emphasised men involvement*, *but they do not come*. *We have asked the women to attend ANC with their husbands*, *especially pregnant women who are tested positive for STIs so we can treat both and counsel them*, *but they would not come*. *Pregnant women with STIs will come back to tell us the men said they do not have any sickness* (What happens to the infected expectant mothers? Do you treat them?). *Yes*, *we treat them*, *but you cannot stop the husbands from infecting them again*. *Therefore*, *we keep on treating STIs in women until they give birth [IDIs*, *other nurses]*.

#### Negative impact of some aspects of ANC on baby

The adult nonpregnant women also had negative perceptions about medicines (folic acid, fesolate, multivariate, calcium and sulphadoxine-pyrimethamine (SP) provided during ANC, thereby discouraging their participation in ANC. In some cases, concerns arose over their resulting in a large baby and a subsequent caesarian section.

Intake of ANC medications will lead to an unprecedented increase in the weight of the unborn child which might prevent them from having normal vaginal childbirth [FGDs, non-pregnant women, Bussie].

The women in the Woggu focus group concurred with this view.

Others were more concerned about negative side effects on them as individuals:

We have pregnant women who complain of vomiting anytime they take it. Others feel dizzy after taking it. I do not take the medicines given. Anytime I took it I vomited everything instantly [FGDs, non-pregnant women, Woggu].

#### Indirect and unnecessary cost imposed on ANC clients

The National Health Insurance Scheme (NHIS) exempts expectant mothers from premium payment and also provides ANC services freely to mothers in Ghana [28]. However, this program is not as effective as expected in the study communities and financial costs remain significant barriers to ANC, particularly in cases of referral to higher level facilities:

Some mothers and families do not honour referrals to next level of care due to financial helplessness [IDIs, other nurses].

This may occur when;

Women who come for ANC and are found to be at risk (sickling positive, HB positive, Hepatitis B positive), need to go through further laboratory investigations, when we asked them to go through further investigations, they refuse because they have no money. So, they will not even attend ANC again until they are in labour [IDIs, other nurses].

When mothers become, unwell and seek care, medicines are sometimes prescribed to them by the doctors/midwives/nurses to buy from chemical sellers (licensed and unlicensed drug dealers); these include medications administered to clients on admission to hospital. In such circumstances, clients weighed the option of seeking care at the health facility and the cost of medicines which are not covered by NHIS:

Each time we go to the health centre or [are] referred to the hospital, medicines were prescribed to us to go and purchase from drug stores. This has made NHIS subscription and renewal of no importance, even going to the hospital, we know they will issue prescription form to us [FGDs, Youth, Charikpong].

Focus group participants indicated that the NHIS now covers only a small portion of the financial burden of the pregnancy related costs:

No, that was then! Now, it only covers patient folder, the hospital bed when on admission and paracetamol tablets. Recently, I received a prescription to buy medicines for my wife (expectant mother), and infusion solutions were extra cost [FGDs, Youth, Charikpong].

#### Health facility factors

Several health facility factors impacted on a willingness to seek ANC. Some of these related to the actual facilities themselves, such as lack of running water, poor lighting, and lack of space to accommodate clients, especially during the rainy days:

We have only one bed each for ANC and delivery. Women sit on the veranda during ANC. Therefore, during rains, some are sent back home because there is no hall to contain all [FGDs, non-pregnant women, Naro/Korinyiri].

Others commented on the negative relationship between nurses and expectant mothers as a barrier to receiving ANC services. Some nurses and midwives acknowledged they both intentionally and unintentionally maltreated pregnant women when providing care. For example, Nadowli hospital is the highest referral facility for the study area, which suggests that the hospital should welcome cases referred there. However, midwives indicated mothers were often treated poorly at the hospital (and at the clinics):

There are many challenges. Challenges from; the client and we the healthcare providers (What are the challenges on the side of the nurses?).

Our communication to expectant mothers are sometimes not good and are either intentional or unintentional. It is due to pressure from the work. For some midwives, it is the pressure. There is the inadequacy of midwives, thereby putting so much pressure on the few. When we are tired, anything the expectant mother does, it irritates the midwife. Other times too we are forced to say something which is not pleasant to clients [IDIs, other nurses].

The midwife explained further that:

We shout at them sometimes when under pressure. However, their lack of resources for childbirth usually contribute to some of the poor attitudes towards them. The up and down movement to save lives makes midwives very tired that, any little thing from the expectant mother may call for insults, unintentionally…. it is not supposed to be that way but because we are stressed [because of] inadequate staff [IDIs, other nurses].

#### Preference for alternative service providers

Diverse opinions were supplied by expectant mothers in regards to their preference for choosing an alternative service provider of care during pregnancy (TBAs and Spiritualists) such as financial constraints (n = 2, 10.5%); family preference (n = 4, 21.1%); preference for TBAs care (n = 5, 26.3%); advice from husband (n = 1, 5.3%); and the fear of caesarean section delivery if attending ANC (n = 1, 5.3%). Mixed reasons constituted 26.3% of explanations.

The FGDs revealed that sociocultural factors often motivated expectant mothers to use alternative sources of prenatal care, particularly their use of local oxytocin” (either direct bark of the tree or burnt into charcoal)” during complications and for self-induction of labour. Demand for this herb operated alongside the other factors already identified, such as financial difficulties, poor services by the NHIS, and unsatisfactory service from health facility staff.

Other mothers utilised maternity care from both mainstream health facility and traditional sources:

When we go to the clinic and are told the baby is in a breech presentation, we go to the TBA, and she can reposition it. Some time ago, my daughter went to Nadowli District hospital and was told she had a breech presentation, and so was booked to consult the doctor the next day. When she returned home, I urged the husband to support her [to] reach the TBA, and it was corrected [FGDs, non-pregnant women, Charikpong].

Many communities raised concerns that participating in ANC would result in delivery at the health care centre, compound or hospital, and subsequent culturally inappropriate disposal of the newborn’s placenta outside the community:

When a child is born at the clinic, the placenta is buried there. Customarily, in Bussie traditional area, it is wrong to bury the placenta in a different community or at the clinic. It should be buried around the family surroundings [FGDs, Opinion leaders, Bussie].

In such cases, delivery by a TBA may be preferred.

#### Lack of awareness of the benefits of ANC by some expectant mothers

Despite the general awareness of the value of ANC across all communities, some pregnant women do not use ANC due to lack of knowledge of its benefits:

In this part of the region, many pregnant women and families are not enlightened, so they do not know the importance of ANC. Some even attend ANC once and never receive care until they give birth at home. Others register and take the card but refuse to attend regularly for the medicines. Midwives are compelled to follow-up [IDIs, other nurses].

## Discussion

This paper explores the extent of ANC use and the reasons underlying the comparatively low level of uptake by expectant mothers. We approached this from perspectives of pregnant women, community residents and health care providers in 8 rural communities.

### The understanding and use of ANC

The findings suggest that the majority of pregnant women and community members had knowledge of the potential positive value of utilising ANC services, including identification of pregnancy problems and their management through skilled and timely interventions at healthcare facilities. This level of knowledge may be attributable to the health education and promotion programmes such as ANC defaulter-tracing, home visits, and child welfare clinics (CWCs) implemented by GHS to ensure increased utilisation of maternity services at a health facility.

However, the majority of expectant mothers who lived in the research area did not commence ANC in their first trimester, thereby limiting midwives’ capacity to make appropriate care to them per the WHO recommended guidelines. This finding supports earlier research undertaken in the study area [2, 4]. However, there was a greater level of registration at ANC compared to other countries [10, 29], and the fact that some women gave birth at the health facility and were interested in getting husbands involved during pregnancy shows an encouraging shift in maternal health care decision-making. These modest improvements may reflect ongoing health promotion works in both rural and urban Ghana [11, 16], as well as other developing regions [30], thereby suggesting the need to intensify health education activities.

### Barriers to use of ANC

Nonetheless, many complex obstacles to the use of ANC exist in the study areas. They fall into four main categories: inaccessibility of ANC, negative perceptions of treatment at health care facilities and perceived side effects of ANC for both the expectant mother and the baby, and lack of family involvement, many of which are underpinned by a diverse array of cultural influences.

Providing financial, emotional and transport assistance to facilitate expectant mother’s ability to access timely and prompt use of ANC is a core theme in maternal health policy. However, non-involvement of family, long distances and poor roads to a health facility, high costs, and cultural and traditional norms premised on the belief that ANC is solely the preserve of women, continue to restrict expectant mothers’ ability to take appropriate maternal health care decisions in the study areas. These attitudes appear to be both culturally and economically motivated, particularly from the perspectives of focus group participants and health professionals. For example, in these agrarian communities many women were prevented from commencing or maintaining ANC follow-ups where ANC schedules coincided with rainfall and the need to keep working in the fields; a finding which corresponds to other research [14]. However, the belief in some communities that it is acceptable for expectant mothers to seek ANC while the husband continues with farm work, that two positive outcomes could be achieved. Such flexibility is necessary given the fact that, the majority of participants identified the cultural inappropriateness of women seeking ANC in the company of their husband/partner. However, given the distance women must travel to utilise maternity services, that lack of involvement by partners (and families) can be both inconvenient and at time life-threatening. A similar study in the UWR found that expectant mothers utilised care only on the community market days due to transport difficulty, although some husbands owned motorbikes [2]. This supports findings in other studies carried out in the same region and Mozambique [31–33]. However, in the Upper East Region of Ghana, family heads consulted soothsayers before deciding on whether to allow the expectant mother to honour the ANC appointment [3].

There are other cultural factors at play here too with some mothers preferring TBA care because they felt their cultural needs could not be met at the health facility setting, particularly if they could be sure that placenta could be buried appropriately. Other culturally premised barriers to prenatal care included stigmatisation of pregnant women by community members, the need for pregnancy rites for prime mothers and concerns over women who “belong to a dangerous belief system” also attending the health facility; in such cases, there seemed little point in receiving ANC. Similar cultural beliefs and practices had a profound impact on the utilisation of skilled maternity services in Papua New Guinea, Kenya and the Upper East Region of Ghana [3, 29, 30].

The perceived side effects of participating in ANC, particularly about taking medications, was identified as another significant barrier. This was supported by other studies [4] that show some expectant mothers will cease the ANC rather than have to take medication. While it is contrary to extensive research showing the value of these medications in achieving improved pregnancy outcomes [5,33], the expectant mother’s reservations were understandable given their beliefs that the medication would not only make them sick but also results in a bigger baby, a complicated labour and therefore the need for a caesarian section. Studies in Uganda and Mozambique found similar issues [31, 32], although these are contrary to the findings in Uganda, where mothers perceived ANC as a programme and thereby failed to utilise it (ANC) if the expectant mother feels healthy.

### Poor nurse-expectant mother relationships

We also found that, poor midwife/nurse-expectant mothers’ relationship was a key barrier to ANC utilisation, with many mothers reporting they were ‘castigated’ by nurses during ANC follow-ups and shown grave disrespect during emergency situations in ways that are contrary to professional codes of conduct and contradict the principles of maternal engagement theory [14, 17]. Women who were maltreated during ANC were less likely to practice the messages communicated to them and less likely to return for follow-up appointments. These results correspond to other research, for example, the poor attitude of nurses influenced the willingness of pregnant women to utilise health facility care in Tanzania [9], while abusive language, violation of the mothers’ privacy and unconsented involvement of other non-professionals during caregiving discouraged adult women elsewhere in Ghana from seeking advice from younger nurses [15]. Overall, the low nurse-client ratio identified as the main cause for nurse stress and negative behaviours has profound consequences for the well-being of both the mother and nurse professionals.

In summary, the findings lend some support to Amzat’s conclusion that women’s lack of freedom to make timely and appropriate decisions concerning their health was one of the leading causes of the inability of pregnant women to utilise skilled maternity care for pregnancy problems [15], under the rights-based theory of decision-making. Furthermore, poor engagement and ownership of the ANC process within the health care setting itself suggest a failure on the part of ANC services to follow the principles of maternal engagement theory [14] and exhibits inadequate attention to cultural sensitivity.

## Conclusion

Despite considerable knowledge regarding the potential benefits of ANC and modest increases in uptake in recent years, there remain significant barriers to accessing and using ANC in practice. While many of these obstacles confirmed those already identified in the literature, we have uncovered other distinctive cultural influences that are significant regional barriers to ANC usage, which have not been found previously in rural Ghana. These obstacles include, for example, placental disposal and concerns over the husband’s involvement during pregnancy and ANC. The findings suggest that antenatal care programs provided by the Ghana Health Service in the local communities should acknowledge and address these cultural distinctions, in addition to more common logistical barriers. Until this is achieved, it is unlikely there would be sustainable and significant improvements in maternal and neonatal health care outcomes in the study areas.

The findings should, however, be read against the backdrop of some limitations. The community specific nature of the study means that the conclusions may not be readily extrapolated to other locations in rural Ghana. Also, two experienced male researchers conducted the focus group discussions. Considering the paternalism currently inherent in the Upper West Region of Ghana, women may have felt intimidated to provide responses that were ‘gender appropriate’ rather than they would have otherwise intended. However, the pragmatism and diversity of the responses by many participants hopefully allay these concerns. Purposive sampling has its own biases about the selection of participants which could influence the results, although we believe this did not happen.

Further investigation into the cultural barriers to utilising antenatal care in the study areas, particularly about service provision and the roles of men in pregnancy is warranted; this is currently in progress. Greater understanding of these issues could facilitate the delivery of more culturally appropriate and empowering ANC, thereby hopefully reduce avoidable causes of maternal morbidities and mortalities.

## Supporting information

S1 File Survey instrument and focus group questions revised. (DOCX)

S2 File Letter _ RHD. (PDF)

S3 File Daffiama bussie issa letter. (PDF)

S4 File Health service assurance. (PDF)
