# Supplementary material for: The use of antenatal care in two rural districts of Upper West Region, Ghana
Source: PLoS One. 2017 Sep 28;12(9):e0185537. doi: 10.1371/journal.pone.0185537 (PMC5619770; doi:10.1371/journal.pone.0185537)
Supplement: S1 File — (DOCX) [file pone.0185537.s001.docx]

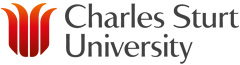


FACULTY OF SCIENCE, SCHOOL OF COMMUNITY HEALTH

ORANGE CAMPUS, NEW SOUTH WALES, AUSTRALIA

QUANTITATIVE SURVEY QUESTIONNAIRE

**RESEARCH TOPIC**: Preparedness for Birth in Rural Areas– Perspectives of Expectant Mothers, Community Residents and Birth Attendants in Two Rural Districts (Nadowli/Kaleo and Daffiama/Bussie/Issa) in Ghana

**PARTICIPANTS: PREGNANT WOMEN**

**Basic Demographic Data**

| **S/N** | **Variable** | | **Response** *(to be circled by interviewer)* | | | **Code** | | **Field Name** | |
| --- | --- | --- | --- | --- | --- | --- | --- | --- | --- |
| **BASIC DEMOGRAPHICS OF PARTICIPANT** | | | | | | | | | |
| 1 | What is the highest level of education you have achieved? | | a) Never attended  b) Primary  c) JHS  d) SHS  e) Tertiary | | | 1  2  3  4  5 | | Educationalstatus | |
| 2 | What is your current marital status? | | a) Single  b) Married  c) Divorced  d) Co-habitation  e) Separated | | | 1  2  3  4  5 | | Maritalstatus | |
| 3 | What is the current job you do for a living? ***(multiple options allowed)*** | | a) Housewife  b) Farmer  c) Wood logging/charcoal burning  d) Local wine brewing  e) Civil Service  f) other (specify) | | | 1  2  3  4  5  6 | | Occupationalstatus | |
| **Obstetric History – Present History** | | | | | | | | | |
| 4 | How old is your current pregnancy? | | a) 1^st^ Trimester  b) 2^nd^ Trimester  c) 3^rd^ Trimester | | 1  2  3 | | | gestationofpregnancy | |
| 5 | Were you on any Family Planning method when you conceived? | | a) Yes  b) No | | 1  2 | | | PregnancyonFP | |
| 6 | IF YES TO Q.5, Did you expect this pregnancy? | | a) Planned  b) Unplanned | | 1  2 | | | Plannedornot | |
| 7 | IF **unplanned**, did you attempt to terminate the pregnancy? | | a) Yes  b) No | | 1  2 | | | Attempttotrminate | |
| 8 | Have you been admitted to hospital for complications in this pregnancy? | | a) Yes  b) No | | 1  2 | | | admissionhistory | |
| 9 | **IF YES TO Q.8;** For what reason/s where you admitted? | | a) Bed rest  b) breech presentation  c) pre-eclampsia (high blood pressure, protein in the urine and swelling of the hands, feet and face)  d) diabetes  e) general observation  f) others (specify) ............ | | 1  2  3  4  5  6 | | | Complicationsinpregnancy | |
| 10 | Have you been on admission on any sickness other than pregnancy? | | a) Yes  b) No | | 1  2 | | | Otheradmissions | |
| 11 | **IF YES TO Q.10,** (*else go to Q.12)* What were you told was the cause of the sickness? | | a) Anaemia  b) Urinary tract infections (e.g. fever, pressure in lower belly, smelly urine, cloudy or reddish urine, back pain)  c) high blood pressure  d) mental health conditions (e.g. sad/low mood, feeling of worthlessness, changes in appetite or sleep or energy, problems thinking/deciding/concentrating, guilt or shameful feeling)  e) **Diabetes (e.g. Gestational diabetes mellitus)**  **f)**  Nausea or vomiting  g) Weight gain in pregnancy/ obesity  h) others (specify) ............... | | 1  2  3  4  5  6  7  8 | | | complicationsinpregnancy | |
| 12 | **IF NO TO Q.10,** Why do you think your pregnancy is staying healthy?  **(*Multiple options allowed*)?** | | a)I do apply the knowledge acquired from ANC lessons  b) I comply to all recommended ANC visits  c) I attend ANC on time  d) I follow other healthy pregnancy advice given  e) I do all recommended tests, scans and immunisations  f) I make advance preparations for early recognition of danger signs and seek early treatment  h) Others (specify) | | 1  2  3  4  5  6  7  8 | | | Reasonsforhealthypregnancy | |
| **Obstetric History –Past History** | | | | | | | | | |
| 13 | How many times have you been pregnant? (**if more than to q.13, continue to q.18;**  **if first pregnancy go to q.34 )** | | | a) One  b) Two  c) Three  d) Four  e) Five and above | | 1  2  3  4  5 | | Gravity | |
| 14 | How many of them were live births? | | | a) One  a) Two  c) Three  d) Four  e) Five and above | | 1  2  3  4  5 | | positiveoutcomesinconceptions | |
| 15 | Did you give birth to the child at your planned place of birth (multipara) **(if no to Q.15 , Continue to Q.16, else move to q.18)** | | | a) Yes  b) No | | 1  2 | | Deliveratplannedplaceofdelivery | |
| 16 | Why could you not give birth at the planned place of child birth? | | | a) Family reason  b) Referral  c) Delay in transport  d) Unable to afford transport fare  e) Not planned  f) No trust in health facility | | 1  2  3  4  5  6 | | Reasonsforinabilitytodeliveratplannedplac | |
| 17 | Where did you have your last child birth? | | | a) CHPS Zone  b) Health Centre  c) District Hospital  d) Private Hospital  e) TBA Home  f) Home  g) on the way to hospital/referral van | | 1  2  3  4  5  6  7 | | Placeofdelivery | |
| 18 | Have you ever had any still birth? (**IF NO TO Q.18, go to Q.21)** | | | a) Yes  b) No | | 1  2 | | Histroyofstillbirths | |
| 19 | **IF YES TO Q.18,** how many have you had in the past **three years (2012-2015)?** | | | a) One  b) Two  c) Three  d) Four  e) Five and above | | 1  2  3  4  5 | | Previousstillbirths | |
| 20 | **IF YES TO Q.18;** Which order did you have the still birth (s) (multiple options allowed) | | | a) First  b) Second  c) Third  d) Fourth and above | | 1  2  3  4 | | Orderofstillbirths | |
| 21 | Have you had any obstetric complications in your recent past pregnancy? **IF “NO” TO Q.21, MOVE TO Q.34)** | | | a) Yes  b) No | | 1  2 | | Previouscomplications | |
| 22 | At what stage and form did it take? **(multiple responses allowed - move to signs on chosen options)** | | | a) Pregnancy  b) Labour & birth  c) Early postnatal | | 1  2  3 | | Stageandformofcomplication | |
| 23 | During pregnancy **(multiple responses allowed)** | | | a) Anaemia  b) Urinary tract infections (e.g. fever, pressure in lower belly, smelly urine, cloudy or reddish urine, back pain)  c) high blood pressure  d) mental health conditions (e.g. sad/low mood, feeling of worthlessness, changes in appetite or sleep or energy, problems thinking/deciding/concentrating, guilt or shameful feeling)  e) **Diabetes (e.g. Gestational diabetes mellitus)**  **f)**  Nausea or vomiting  g) Weight gain in pregnancy/ obesity  h) others (specify) ............ | | 1  2  3  4  5  6  7  8 | | Complicationsinpregnancy | |
| 24 | During Labour and delivery ***(multiple responses allowed)*** | | | a) Profuse vaginal bleeding  b) Prolonged labour longer than 12 hrs  c) Convulsions  d) Retained placenta  e) General weakness or collapse  f) Other …………… | | 1  2  3  4  5  6 | | Complicationsinlabour | |
| 25 | Early Postpartum ***(multiple responses allowed)*** | | | a) Severe vaginal bleeding  b) Foul smelling discharge  c) High fever  d) Abdominal pain  e) Other …………….. | | 1  2  3  4  5 | | Complicationsinearlypospartum | |
| 26 | When did you first realise there might be a problem in the pregnancy? | | | a) during early warning signs  b) it had already began  c) when on admission at health facility  d) upon meeting a health volunteer  e) during antenatal  f) other (specify) ......... | | 1  2  3  4  5  6 | | Realiseproblem | |
| 27 | How did the problem start? | | | a) after carrying head load  b) after brewing local wine  c) when I returned from farm labour  d) after attending a funeral  e) when I went to burn charcoal  f) after felling and/or fetching firewood  g) other ......... | | 1  2  3  4  5  6  7 | | Originofproblem | |
| 28 | Have you had any previous pregnancy complications which led to miscarriage? | | | a) Yes  b) No | | 1  2 | | Pastcomplications leadingtomiscarriage | |
| 29 | Which pregnancy in the order did you have these complications? | | | a) First  b) Second  c) Third  d) Fourth and above | | 1  2  3  4 | | Orderofpregnancywithcomplications | |
| 30 | What complications did you experience in the order mentioned above (Q.32)? ***(multiple responses allowed)*** | | | a) Abdominal pains  b) Fever  c) headache  d) blurred vision  e) bleeding  f) others | | 1  2  3  4  5  6 | | Complicationsinfirstpregnancy | |
| 31 | What form did it take? ***(multiple responses allowed)*** | | | a) Abdominal pains  b) Fever  c) Headache  d) Blurred vision  e) Bleeding  f) Depression  g) others................... | | 1  2  3  4  5  6  7 | | Natureofpastcomplications | |
|  | **IF NON- PRIMIGRAVIDA:** | | |  | |  | |  | |
| 32 | Was your most recent child birth Normal or Caesarean section? | | | a)Normal – V birth  b)Normal – CS  c)Emergency – V birth  d)Emergency - CS | | 1  2  3  4 | | Kindofdelivery | |
| 33 | If Emergency –CS,  What problems did you encounter after child birth? ***(multiple responses allowed)*** | | | a)Difficulty securing Blood donor  b)Severe bleeding  c) Disability ……………..  d) Blurred vision  e) Infections  f) Other .......... | | 1  2  3  4  5  6 | | Problemsinemergencydelivery | |
| **Socioeconomic** | | | | | | | | | |
| 34 | What is your average income per month? | | a)GHS 10-50  b)GHS51-100  c)GHS101-200  d)GHS200 & above | | | 1  2  3  4 | | Incomelevel | |
| 35 | Do you receive some forms of support from family during pregnancy? | | a) Yes  b) No | | | 1  2 | | Receivefamilysupport | |
| 36 | What form does the family support take? ***(multiple responses allowed)*** | | a) Assistance to ANC  b) Financial provision to buy essential medicines  c) Financial support to do lab. tests  d) Financial support to renew health insurance  e) Purchase of birth kits  f) Money for complications/emergency | | | 1  2  3  4  5  6 | | Formoffamilysupport | |
| 37 | Is there any other support you need or would like from your family? **(IF YES to Q. 37, continue to next question, otherwise go to Q.39)** | | a) Yes  b) No | | | 1  2 | | Othersupport | |
| 38 | What other forms of support do you need from your family? ***(multiple options allowed)*** | | a) emotional support  b) counselling from health professional  c) psychological support from husband  d) sense of love from husband  e) anti-depressants  f) Religious counselling | | | 1  2  3  4  5  6 | | Natureofsupportfromfamily | |
| **Transportation** | | | | | | | | | |
| 39 | Which sub-district are you coming from? | | a) Nadowli  b) Charikpong  c) Takpo  d) Dapuori  e) Kaleo  f) Jang  g) Sombo  h) Nanvilli  i) Daffiama  j) Bussie  k) Fian  l) Kojokpere  m) Issa | | | 1  2  3  4  5  6  7  8  9  10  11  12  13 | | Attendancefrom Sub-district | |
| 40 | Is there any transport (*trotro* services) service available to your community? | | a)Yes  b) No | | | 1  2 | | Transportavailable | |
| 41 | **IF NO TO Q.40** What is your main regular mode of travel to health facility? | | a)by motorbike  b)motorking/tricycle  c)on foot  d) by bicycle  e) only when bus comes to the community | | | 1  2  3  4  5 | | Mainmodeoftravel | |
| 42 | Is there a community vehicle for pregnancy emergency? | | a) Yes  b) No | | | 1  2 | | Communitytransport | |
| 43 | Which type of transport do you mostly use when accessing maternal health service? | | a) Public transport (trotro)  b) Private car  c) Motorbike  d) Motorking  e) Walk | | | 1  2  3  4  5 | | Modeoftransport | |
| 44 | How many vehicle transfers do you make to access the nearest or most relevant health facility? | | a) One transfer  b) Two transfers  c) Three transfers  d) Four transfers  e) Straight/direct means | | | 1  2  3  4  5 | | Transittrips | |
| 45 | What is the state of roads linking your community to health facility? | | a) motorable all year round by car  b) not motorable year round by car  c) accessible by motorbike only all year round  d) not accessible by motorbike all year round | | | 1  2  3 | | Stateofroads | |
| 46 | Rate relevance of poor state of vehicles as maternal hazard | | a) Very important  b) Important  c) Average  d) Not at all | | | 1  2  3  4 | | Vehiclehazzard | |
| 47 | What is the dominant means of transport during pregnancy complications? | | a) Public transport (trotro)  b) Private car  c) Motor bike  d) Motorking/tricycle  e) Ambulance | | | 1  2  3  4  5 | | Emergencytransport | |
| 48 | In case of obstetric complications, by what means do you make phone calls or reach out to health facility? | | a) Through public phone booth network system  b) Personal mobile phone  c) Sending for a health staff member  d) Other (specify) | | | 1  2  3  4 | | Emergencyphonecall | |
| **Use of Antenatal Care** | | | | | | | | | |
| 49 | Are you currently receiving (using/accessing) antenatal care? | | a) Yes  b) No | | |  | | ReceivingandusingANC | |
| 50 | What care do the nurses provide you with during your visits? (**multiple options allowed**) | | 1. Monitoring blood pressure and signs of pre-eclampsia/eclampsia 2. PMTCT STIs prevention 3. Iron/folate supplementation 4. Treated bednets distribution 5. Measure weight/body mass index 6. Risks detection 7. Tetanus toxoid immunisation 8. Birth preparedness/CR 9. Others (specify) | | | 1  2  3  4  5  6  7  8  9 | | Contentofcareprovided | |
| 51 | Do the nurses give you lessons during the visits? | | 1. Yes 2. No | | | 1  2 | | LessonsatANC | |
| 52 | Is the content of the lessons helpful to your health and pregnancy? | | a) Yes  b) No | | | 1  2 | | relevanceofANClessons | |
| 53 | Which week did you commence antenatal? | | a) 4 weeks  b) 5 weeks  c) 6 weeks  d) 7 weeks  e) 8 weeks and above | | | 1  2  3  4  5 | | Firstantenatalvisit | |
| 54 | **(IF NO TO Q.52; if YES, go to Q.55)** What prevents you from attending antenatal care? ***(multiple options allowed***) | | a) Distance to health facility  b) Cultural beliefs such as........  c) Could not afford essential medicines  d) Poor attitude of nurses towards clients  e) Religious Reasons  f) Others (Specify) | | | 1  2  3  4  5  6 | | Reasonfornonattendance | |
| 55 | Do you seek maternal health care services from other sources? | | 1. Yes 2. No | | | 1  2 | | Sourcesforcare | |
| 56 | **If YES TO Q.55, IF No, go to Q.58** from whom do you seek these services? ***(multiple responses allowed)*** | | a) TBA  b) Spiritualist/Pastors  c) Native Doctors  d) Herbalist  e) Other……………… | | | 1  2  3  4  5 | | Othersourcesofcare | |
| 57 | Why do you prefer TBAs or others to antenatal health care? ***(multiple options allowed)*** | | a) Financial constraints  b) Family preference  c) Negative attitude from nurses  d) TBA for massage  e) Advice from husband  f) fear of caesarean section  g) Church for spiritual attention  h) Others (specify) | | | 1  2  3  4  5  6  7  8 | | reasonsforpreferenceofTBAs | |
| 58 | Does your religion speak against skilled male attendance during care seeking and birth? | | a) Yes  b) No | | | 1  2 | | Religiousinfluence | |
| 59 | **IF “YES” to Q.58:** Why do you seek antenatal care services? ***(multiple options allowed***) | | a) Protect child’s life  b) For easy child birth  c) Protect mother’s life  d) Protect both child &mother’s life  e) To identify any health problems during the pregnancy | | | 1  2  3  4  5 | | Reasonsforanc | |
| 60 | Where did you have your last child birth? | | a) Home – TBA  b) Home – Relatives  c) Hospital/Health centre- SBA  d) Others (specify) | | | 1  2  3  4 | | Placeofdelivery | |
| 61 | How many visits did you make to antenatal care in your immediate past pregnancy? (**If less than 4 visits,** **continue** **to Q.62)** | | a) One visit  b) Two visits  c) Three visits  d) Four or more visits  e) Never attended | | | 1  2  3 | | Numberofpastancattendance | |
| 62 | How many visits have you made to antenatal care in your current pregnancy? (**If less than 4 visits,** **continue** **to Q.63)** | | a) One visit  b) Two visits  c) Three visits  d) Four or more visits  e) Never attended | | | 1  2  3 | | PresentANCattendance | |
| 63 | Why could you not make **4 or more** visits | | a)Distance to ANC venue  b)Cultural reasons e.g. family objections  c)Visited TBA  d)Negative attitude from nurses  e)Transport challenge  f) Social reasons e.g. funeral  g) not far enough along in pregnancy  h) other (please specify) | | | 1  2  3  4  5  6  7  8 | | Reasonsforlessthanfourvisisits | |
| 64 | What do you think/perceive to be the main purposes of antenatal care? | | a) only receive tetanus injection  b) iron folic acid tablets distribution point  c) a place nurses yell at clients  d) a social gathering  e) other ……….. | | | 1  2  3  4  5 | | perceptionaboutANC | |
| 65 | What other benefits do you think the antenatal health care offer you? | | a)Healthy pregnancy classes/lessons  b)Preventive measures through including immunisations throughout the period  c)Health education and promotion for you and family  d)Recognition and management of pregnancy and related complications  e)Prepare us emotionally and physically for the pregnancy  f) others (specify) | | | 1  2  3  4  5  6 | | OtherperceivedbenefitsofANC | |
| **Knowledge of Basic Components of BPCR** | | | | | | | | | |
| 66 | Have you made any preparations for safe pregnancy and delivery( **IF no, to Q.58 go to Q.61**) | a) Yes  b) No | | | | | 1  2 | | Madepreparation |
| 67 | **IF “YES” TO Q.66**: Can you mention the kinds of preparations you have done or are doing for a safe pregnancy and delivery? ***(multiple response allowed)*** | a) Saving money for use in emergencies or during labour  b) Plan/Preparations for place of birth  c) Identifying transport in case of complications and during labour  d) Identifying a birth companion  e) Identifying a blood donor  f) Identifying Skilled attendant  g) buy birth kit | | | | | 1  2  3  4  5  6  7 | | componentsofBPCR |
| 68 | Are there any other preparations for safe pregnancy and delivery that you still plan to do?***(mention them; multiple response allowed)*** | a) Saving money for use in emergencies or during labour  b) Plan/Preparations for place of birth  c) Identifying transport in case of complications and during labour  d) Identifying a birth companion  e) Identifying a blood donor  f) Identifying Skilled attendant  g) buy birth kit  h) other (specify) | | | | | 1  2  3  4  5  6  7  8 | | Otherpreparationsforsafepregnancy |
| 69 | Where do you get information about birth/complication preparedness? | a) Nurses  b) family members  c) friends  d) local media  e) others (specify) | | | | | 1  2  3  4  5 | | Adviceonpreparedness |
| 70 | Do you follow the advice of the nurses or choose your own way? | a)Take to their advice  b) Choose my own way | | | | | 1  2 | | Reactiontoadvicefromnurses |
| 71 | (**IF OPTION “b”, to Q. 70)** Why do you choose your own way? | a)do not trust their ideas  b) thought it is for those with complications  c) communicated to me with no respect  d) social/cultural values  e) heeded to the advice from TBAs  f) Other (Specify) | | | | | 1  2  3  4  5  6 | | Reasonfornottakingtoadvice |
| 72 | Do you discuss with family members about interventions in BPCR? | a)Yes  b)No | | | | | 1  2 | | discusswithfamilyonBPCRinterventions |
| 73 | Which area of support do you receive support from family members? | a) danger signs  b) where to give birth  c) finance arrangement  d) arrangement for transport  e) arrangement of blood donor  f) accompanying person  g) no discussion with family members | | | | | 1  2  3  4  5  6  7 | | Supportfromfamilymembers |
| 74 | Who accompanies you to health facility during ANC/Complications? | a) Husband  b) Mother-in-law  c) Mother  d) Close relatives | | | | | 1  2  3  4 | | Supporttofacility |

75. Are there some other issues you want me to know about pregnancy and complications in this community?

____________________________________________________________________________________________________________________________________________________________________________________________________________________________________________________________________________________________________________________________________________________________________________________________________________________________________________________________________________________________________________

76. What other information will like me to pass on to the government for improved maternal and neonatal health in your community?

____________________________________________________________________________________________________________________________________________________________________________________________________________________________________________________________________________________________________________________________________________________________________________________________________________________________________________________________________________________________________________

77. In your own words, what can you say about the behaviour of nurses towards pregnant women during antenatal?

____________________________________________________________________________________________________________________________________________________________________________________________________________________________________________________________________________________________________________________________________________________________________________________________________________________________________________________________________________________________________________

78. What suggestions do you want make for better antenatal services in your area?

____________________________________________________________________________________________________________________________________________________________________________________________________________________________________________________________________________________________________________________________________________________________________________________________________________________________________________________________________________________________________________

**Thank you for participating in the survey.**


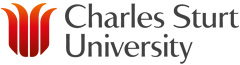


FACULTY OF SCIENCE, SCHOOL OF COMMUNITY HEALTH

ORANGE CAMPUS, NEW SOUTH WALES, AUSTRALIA

QUANTITATIVE SURVEY QUESTIONNAIRE

**RESEARCH TOPIC**: Preparedness for Birth in Rural Areas– Perspectives of Expectant Mothers, Community Residents and Birth Attendants in Two Rural Districts (Nadowli/Kaleo and Daffiama/Bussie/Issa) in Ghana

**PARTICIPANTS: HEALTH FACILITY HEADS**

*Your response to this questionnaire will serve as source of information to the research paper for academic purpose. Any response you provide here is strictly confidential and will be used exclusively for the research purpose. Your honesty in giving exact information is vital for the research outcome - improved maternal and neonatal health, to be reliable.*

**What are the categories of staff in your facility?**

(Complete the table on the Staffing Capacity of the Health Facility)

| S/N | Category | No. at Post | Male | Female | No. Required |
| --- | --- | --- | --- | --- | --- |
| 1. | Physician Assistants |  |  |  |  |
| 2. | Public Health Nurse |  |  |  |  |
| 3. | Midwives |  |  |  |  |
| 4. | Nutrition Officers |  |  |  |  |
| 5. | Registered General Nurses |  |  |  |  |
| 6. | Community Health Nurses |  |  |  |  |
| 7. | Enrolled Nurses |  |  |  |  |
| 8. | Record Assistants |  |  |  |  |
| 9. | Health Promotion Officers |  |  |  |  |
| 10. | Field Technicians |  |  |  |  |
| 11. | Mental Health Officers |  |  |  |  |
| 12. | Health Information Officers |  |  |  |  |
| 13. | Ambulance Drivers |  |  |  |  |

Obstetric Information and General Status of Preparedness

| **S/N** | **Variable** | **Response** | **Code** | **Field Name** |
| --- | --- | --- | --- | --- |
| 1 | Does the facility receive emergency referrals on pregnancy complications from communities? | a)Yes  b)No | 1  2 | Receiveemeergencyreferralsinpregnancy |
| 2 | Which areas in the sub-district do you mostly receive emergency obstetric cases from? | a) Very remote parts  b) nearby areas  c) all parts of the district | 1  2  3 | Majorreferralplaces |
| 3 | What is your primary means for emergency referrals? | a)Facility ambulance  b)National ambulance service  c) Client -arranged means  d) Facility van  e) Public Transport  f) Other | 1  2  3  4  5  6 | meansusedduringreferrals |
| 4 | Are there some cultural beliefs in these communities that affect save pregnancy management? | a)Yes  b) No | 1  2 | Culturalbeliefsinpregnancies |
| 5 | If yes, what are the most important cultural issues? (*multiple responses allowed*) | a)meal exclusion e.g. egg/meat intake will make child witch  b) taking shower at night will infest the baby  c) pregnant women should not eat meat, will be tagged as witch  d) no spicy food intake in pregnancy  e) must take traditional herbs “mansugo”  f) must not eat beans related food | 1  2  3  4  5  6 | culturalissuesinpregnacy |
| 6 | If YES, what efforts have the facility and the community made about it? | a)Mass community sensitisation  b) Education during ANC  c) Targeted public health education | 1  2  3 | effortsonawarenesscreation |
| 7 | Do some pregnant women still patronise TBAs services? | a) Yes  b) No | 1  2 | PatronageofTBAs |
| 8 | Have you ever had obstetric cases within the past two years (January, 2013- December, 2015)? | a) Yes  b) No | 1  2 | Obstetriccases |
| 9 | If Yes, how many on the average do you record per week times? | a)one case  b) two case  c) three cases  d) four cases  e) five cases and above | 1  2  3  4  5 | Numberofobstetriccases |
| 10 | How many of these cases required basic and or comprehensive care? | a)basic ………  b)comprehensive ……… | 1  2 | obstetriccasegrouping |
| 11 | Does the facility contain all required skilled personnel to handle both BEmoNC and CEmoNC? | a)Yes  b)No | 1  2 | capacitytohandleEmONCcases |
| 12 | What were the outcomes of those cases? (multiple responses allowed) | a) Referral  b) Still births  c) Loss of women  d) Miscarriage  e) Successful delivery? | 1  2  3  4  5 | Outcomesofobstetriccases |
| 13 | Do you have adequate equipment to handle pregnancies? | a)Yes  b)No | 1  2 | Equipmentsavailability |
| 14 | What advice do you render to clients during ANC on BPCR? (multiple options allowed) | a)Danger signs  b)Where to go for care  c)Place of birth  d)Transport arrangement  e)Finance  f) Blood donor  g) other (specify) | 1  2  3  4  5  6  7 | contentofANCservices |
| 15 | Has your facility conducted any EmONC in the past one year? | a)Yes  b)No | 1  2 | EmoNCServices |
| 16 | Which of these BEmONC services have you conducted? | a) Administering antibiotics, uterotonic drugs (oxytocin) and anticonvulsants (magnesium sulphate)  b) Manual removal of the placenta  c) Removal of retained products following miscarriage or abortion  d) Assisted vaginal birth, preferably with vacuum extractor;  e)Basic neonatal resuscitation care  f) other ............... | 1  2  3  4  5  6 | BEmONCServicesrendered |
| 17 | Do you conduct these CEmoNC services in your facility? | a) blood transfusion  b) performing caesarean sections  c) care to sick and low-birth weight newborns and resuscitation  d) other ......... | 1  2  3  4 | CEmONCservicesrenderd |
| 18 | Do you have all the required staff and supplies to carry out this service? | a) Yes  b) No | 1  2 | Staffneededforobstetricservices |
| 19 | Have you failed in conducting any of these BEmONC services? | a) Yes  b) No | 1  2 | outcomesofBEmONCservices |

20. What are the causes of maternal deaths recorded in your facility (*the table is ONLY a guide)*

| **S/N** | **Period/Cause** | **2012** | **2013** | **2014** |
| --- | --- | --- | --- | --- |
|  |  | **Maternal deaths** | | |
| 1 | Haemorrhage |  |  |  |
| 2 | Preeclampsia |  |  |  |
| 3 | Infection |  |  |  |
| 4 | Sepsis |  |  |  |
| 5 | Caesarean Section error |  |  |  |
| 6 | Prolonged labour |  |  |  |
| 7 | Inadequate obstetric care |  |  |  |
| 8 | Indirect causes |  |  |  |
| 9 | Ante partum |  |  |  |
| 10 | Postpartum |  |  |  |
| 11 | Others............. |  |  |  |

21. What are the causes of still births in the Catchment Area of the facility?

| S/N | Cause/Period | 2012 | 2013 | 2014 |
| --- | --- | --- | --- | --- |
| 1 | bacterial infection |  |  |  |
| 2 | Birth asphyxia |  |  |  |
| 3 | birth defects, especially pulmonary hypoplasia |  |  |  |
| 4 | chromosomal aberrations |  |  |  |
| 5 | growth retardation |  |  |  |
| 6 | Induced Foetal Demise |  |  |  |
| 7 | intrahepatic cholestasis of pregnancy |  |  |  |
| 8 | maternal diabetes |  |  |  |
| 9 | high blood pressure, including preeclampsia |  |  |  |
| 10 | maternal consumption of recreational drugs (such as alcohol, nicotine, etc.) or pharmaceutical drugs contraindicated in pregnancy |  |  |  |
| 11 | postdate pregnancy |  |  |  |
| 12 | placental abruptions |  |  |  |
| 13 | physical trauma |  |  |  |
| 14 | Others (specify) |  |  |  |

22. What equipment do you use during pregnancy management- antenatal, labour, birth and post-birth care?

[ *List the WHO/MoH/GHS recommended equipments for safe management of pregnancy complications through to post-delivery (Labour/Birth Suppliers/Equipment Capacity Assessment)*]

| S/N | Name of Equipment | Year supplied | Quantity in Use (good condition) | Quantity not in Use (poor condition) | Quantity required |
| --- | --- | --- | --- | --- | --- |
|  |  |  |  |  |  |
|  |  |  |  |  |  |
|  |  |  |  |  |  |
|  |  |  |  |  |  |
|  |  |  |  |  |  |
|  |  |  |  |  |  |
|  |  |  |  |  |  |
|  |  |  |  |  |  |
|  |  |  |  |  |  |
|  |  |  |  |  |  |

23. What maternal and newborn intervention programmes do you run in the catchment area of your facility?

| S/N | Maternal and newborn health programs | Duration | Source of funding | Location |
| --- | --- | --- | --- | --- |
|  |  |  |  |  |
|  |  |  |  |  |
|  |  |  |  |  |
|  |  |  |  |  |
|  |  |  |  |  |
|  |  |  |  |  |
|  |  |  |  |  |

24. What is the average distance of the communities within your sub-district to the facility?

*(List the Communities and their Average Distances to the nearest Health Facility)*

| Name of Study Community | Av. Distance to nearest facility | Av. Distance to nearest referral hospital |
| --- | --- | --- |
|  |  |  |
|  |  |  |
|  |  |  |

25. What sexual reproductive cultural beliefs are practiced in these communities?

__________________________________________________________________________________________________________________________________________________________________________________________________________________________________________________________________________________________________________________________________________________________________________________________________________________________

26. What challenges does the facility experience in executing health education relating to maternal and new-born health?

__________________________________________________________________________________________________________________________________________________________________________________________________________________________________________________________________________________________________________________________________________________________________________________________________________________________

27. What institutional challenges are you facing in your carrying out maternal and new-born health programmes?

________________________________________________________________________________________________________________________________________________________________________________________________________________________________________________________________________________________________________________________________________

28. To what extent do community leaders assist during any intervention programmes implemented by the facility?

__________________________________________________________________________________________________________________________________________________________________________________________________________________________________________________________________________________________________________________________________________________________________________________________________________________________

29. Are there any local initiatives by the community to prevent maternal and still births? If yes, please give details of those programs.

____________________________________________________________________________________________________________________________________________________________________________________________________________________________________________________________________________________________________________________________________________________________________________________________________________________________________________________________________________________________________________

30. What suggestions can you make to help improve maternal and new-born outcomes in this district?

**__________________________________________________________________________________________________________________________________________________________________________________________________________________________________________________________________________________________________________________________________________________________________________________________________________________________**

***Thank you for participating in the survey!***


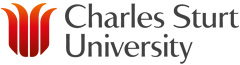


FACULTY OF SCIENCE, SCHOOL OF COMMUNITY HEALTH

ORANGE CAMPUS, NEW SOUTH WALES, AUSTRALIA

QUALITATIVE DISCUSSION GUIDE (**FOCUS GROUPS DISCUSSIONS)**

**RESEARCH TOPIC**: Preparedness for Birth in Rural Areas– Perspectives of Expectant Mothers, Community Residents and Birth Attendants in Two Rural Districts (Nadowli/Kaleo and Daffiama/Bussie/Issa) in Ghana

**PARTICIPANTS: NON-PREGNANT WOMEN**

1. What are the causes of maternal deaths in your community?

2. What are the causes of Neonatal deaths in your community?

3. What things/activities should pregnant women do in your community?

4. What activities (cultural, social, economic) and general daily lifestyle should pregnant women not do in your community?

5. What are the things women should do to stay healthy during pregnancy?

6. If someone talked to you about birth preparedness and being ready for complications in pregnancy, what do you think they would be talking about?

7. What can you tell me about community initiatives towards ensuring safe motherhood?

8. What can you tell me about other settings (places) or groups where pregnancy related matters are talked about in the community?

9. What forms of support do these groupings offer to pregnant women?

10. What sorts of things does your family do (or do families do) to help ensure safe pregnancy and child birth?

11. What reproductive cultural practices exist in your communities?

12. a. what do you think stops women from seeking antenatal care?

12. b. What do you think stops women from wanting to give birth in a health facility?

13. What do you think are the best ways to prevent maternal deaths and still births in the community?

14. What will you suggest should be done to prevent pregnant women and new-borns from dying?

***Thank you for participating in the discussion!***


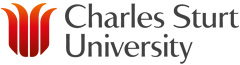


FACULTY OF SCIENCE, SCHOOL OF COMMUNITY HEALTH

ORANGE CAMPUS, NEW SOUTH WALES, AUSTRALIA

QUALITATIVE INTERVIEW GUIDE (**FOCUS GROUPS DISCUSSIONS)**

**RESEARCH TOPIC**: Preparedness for Birth in Rural Areas– Perspectives of Expectant Mothers, Community Residents and Birth Attendants in Two Rural Districts (Nadowli/Kaleo and Daffiama/Bussie/Issa) in Ghana

**PARTICIPANTS: YOUTH**

1. If someone talked to you about birth preparedness and being prepared for complications in pregnancy, what do you think they would be talking about?

2. What are your perceptions of safe pregnancy?

3. What are the various kinds of support pregnant women receive from family members?

4. What community plans are there for emergency situations in pregnancy?

5. What reproductive cultural practices exist in the community?

6. How do you think these practices have some effects on pregnancy outcomes?

7. What do you think can be done in the community to improve the health of pregnant women and newborns?

***Thank you for participating in the discussion!***


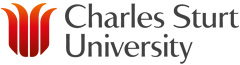


FACULTY OF SCIENCE, SCHOOL OF COMMUNITY HEALTH

ORANGE CAMPUS, NEW SOUTH WALES, AUSTRALIA

QUALITATIVE INTERVIEW GUIDE (**FOCUS GROUPS DISCUSSIONS)**

**RESEARCH TOPIC:** Preparedness for Birth in Rural Areas– Perspectives of Expectant Mothers, Community Residents and Birth Attendants in Two Rural Districts (Nadowli/Kaleo and Daffiama/Bussie/Issa) in Ghana

**PARTICIPANTS: OPINION LEADERS**

1. What kinds of things/activities should a woman do when pregnant?

- prompts – at home? – at work? – in the community

2. What activities (cultural, social, economic) should pregnant women not do in your community?

3. What are the things women should do to stay healthy during pregnancy?

4. What do you think are the main causes of deaths of pregnant women or after delivery in this community/district?

5. What do you think are the main causes of deaths of newborns in this community/district?

6. If someone talked to you about birth preparedness and being ready for complications in pregnancy, what do you think they would be talking about?

7. What can you tell me about community initiatives towards ensuring safe motherhood?

8. What can you tell me about other settings (places) or groups where pregnancy related matters are talked about in the community?

9. What forms of support do these groupings offer to pregnant women?

10. What sorts of things does your family do (or do families do) to help ensure safe pregnancy and child birth?

11. What reproductive cultural practices exist in your communities?

12. a. what do you think stops women from seeking antenatal care?

b. What do you think stops women from wanting to give birth in a health facility?

13. What do you suggest are the roles of men in pregnancy?

14. Are men active as expected in supporting the woman during the period of pregnancy?

15. What do you think are the best ways to prevent maternal deaths and still births in the community?

***Thank you for participating in the discussion!***


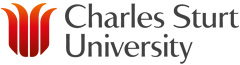


FACULTY OF SCIENCE, SCHOOL OF COMMUNITY HEALTH

ORANGE CAMPUS, NEW SOUTH WALES, AUSTRALIA

QUALITATIVE INTERVIEW GUIDE (OTHER NURSES)

**RESEARCH TOPIC:** Preparedness for Birth in Rural Areas– Perspectives of Expectant Mothers, Community Residents and Birth Attendants in Two Rural Districts (Nadowli/Kaleo and Daffiama/Bussie/Issa) in Ghana

**OTHER NURSES**

1. What arrangements are put in place for pregnancy/birth emergency referrals?

2. What is you perception on why some pregnant refusal to use antenatal care services or complete the recommended number of visits?

3. Do you think clients’ physical and social integrity are compromised when nurses/midwives are carrying out their duty of care?

4. What is your broad view about the challenges midwives face in carrying out duty of care to clients (antenatal care, women in labour or postnatal care)?

5. In your opinion, what recommendations will you make for improved maternal and neonatal health outcomes in the rural/hard-to-reach areas?

***Thank you for participating in the interview!***
