# Supplementary material for: The use of antenatal care in two rural districts of Upper West Region, Ghana
Source: PLoS One. 2017 Sep 28;12(9):e0185537. doi: 10.1371/journal.pone.0185537 (PMC5619770; doi:10.1371/journal.pone.0185537)
Supplement: S2 File — (PDF) [file pone.0185537.s002.pdf]

ORG-03/F-01

In case of the reply the number and date of this letter should be quoted.

GHANA HEALTH SERVICE
REGIONAL HEALTH ADMIN
P. O. BOX 298
WA UWR
GHANA

My Ref. No GHS/UWR/ER-17-51
Your Ref. No...............

January 09, 2017

Tel: +233 07 56 22 204 or 22 016
Fax: +233 07 56 22 471
Email: ghs-uwr@africaonline.com.gh

THE DDHS, DBI
THE DDHS, NADOWLI – KALEO

INTRODUCTORY LETTER: JOSHUA SUMANKUURO

The bearer of this letter is to embarked on a doctoral research project on preparedness for birth in rural areas and the perspectives of expectant mother, community residents and birth attendants in two rural districts.

Kindly accord him the necessary support and cooperation and take the necessary steps to ensure that the privacy and confidentiality of staff and clients who will be participating in the study are guaranteed.

Thank you.

BASADI RICHARD
DEPUTY CHIEF HEALTH RESEARCH OFFICER
FOR: REGIONAL DIRECTOR OF HEALTH SERVICES

Cc: Research file
Mr Joshua Sumankuuro

Seen
All Concerned units (RCH/DC
to not and support studen
12-1-17
