# Supplementary material for: The use of antenatal care in two rural districts of Upper West Region, Ghana
Source: PLoS One. 2017 Sep 28;12(9):e0185537. doi: 10.1371/journal.pone.0185537 (PMC5619770; doi:10.1371/journal.pone.0185537)
Supplement: S3 File — (PDF) [file pone.0185537.s003.pdf]

(CENTRAL ADMINISTRATION DEPARTMENT)

In case of reply the date and number of this letter should be quoted

Our Ref.DBIDA/.......

Your Ref...........

Tel.

Email: daffbussieissa@yahoo.com.

Republic of Ghana

Post Office Box 2,

Issa - Wa,

Upper West Region.

Date: 4th November, 2015

LETTER OF ASSURANCE

MR. JOSHUA SUMANKUURO

The purpose of this letter is to inform you that the management of the Daffiama-Bussie-Issa District have granted Mr. Joshua Sumankuuro permission to conduct the research titled "**Preparedness for Birth in Rural Areas– Perspectives of Expectant Mothers, Community Residents and Birth Attendants in Two Rural Districts in Ghana**", a student of Charles Sturt University, Australia. This also serves as assurance that this study complies with the ethics conditions in Ghana.

He shall be permitted collect from any employee or citizen including the pregnant women, subject to their personal consent. It is my fervent hope that this request will be granted him.

Thank you.

....

HON. FIDELIS N. ZUMAKPEH
DISTRICT CHIEF EXECUTIVE
DAFFIAMA/BUSSIE/ISSA
DISTRICT
ISSA

The Executive Officer
Human Research Ethics Committee
Office of Academic Governance
Charles Sturt University
Panorama Avenue
Bathurst NSW 2795
Tel: (02) 6338 4628
Email: ethics@csu.edu.au
