# Supplementary material for: The use of antenatal care in two rural districts of Upper West Region, Ghana
Source: PLoS One. 2017 Sep 28;12(9):e0185537. doi: 10.1371/journal.pone.0185537 (PMC5619770; doi:10.1371/journal.pone.0185537)
Supplement: S4 File — (PDF) [file pone.0185537.s004.pdf]

In case of reply the date and number of his letter should be quoted<br><br>*Tel: +233 (03920) 91069*<br>*Fax:*<br>*Email:*<br><br>*My Ref. No GHS/NDHD/PS-7*<br>*Your Ref…*<br>***Our Core Values:*** *People Centered Professionalism, Team Work, Innovation, Discipline and Integrity.*	GHANA HEALTH SERVICE<br>GHS<br>Your Health • Our Concern	*GHANA HEALTH SERVICE*<br>DISTRICT HEALTH DIRECTORATE<br>*P. O. BOX 3,*<br>*NADOWLI. UWR.*<br>GHANA<br><br>9th November, 2015.

The Executive Officer
Human Research Ethics Committee
Office of Academic Governance
Charles Sturt University
Panorama Avenue
Bathurst NSW 2795
Tel: (02) 6338 4628
Email: ethics@csu.edu.au

Dear Sir/Madam

PERMISSION GRANT NOTIFICATION
MR. JOSHUA SUMANKUURO

On behalf of the District Health Administration, I write to formally indicate our awareness of the Research on "**Preparedness for Birth in Rural Areas– Perspectives of Expectant Mothers, Community Residents and Birth Attendants in Two Rural Districts in Ghana",** a student of Charles Sturt University, Australia.

We are aware that Joshua intends to conduct his research by administering a survey questionnaire to our employees and pregnant women using our service (subject to their personal consent), which will enable him obtain an understanding of issues pertaining in birth preparedness and complication readiness in our rural and remote areas.

By this letter, I affirm that we shall provide relevant information to support the conduct of his study.

Thank you.

FLORENCE ANGSOMWINE
DIRECTOR OF HEALTH SERVICES
NADOWLI/KALEO DISTRICT
NADOWLI

DISTRICT DIRECTOR
GHANA HEALTH SERVICES
BOX 3, NADOWLI - UWR
